# Polymeric biocompatible iron oxide nanoparticles labeled with peptides for imaging in ovarian cancer

**DOI:** 10.1042/BSR20212622

**Published:** 2022-02-11

**Authors:** Deepshikha Shahdeo, Akanksha Roberts, Veerbhan Kesarwani, Milena Horvat, Raghuraj Singh Chouhan, Sonu Gandhi

**Affiliations:** 1 DBT–National Institute of Animal Biotechnology (DBT-NIAB), Diagnostic Division, Hyderabad 500032, Telangana, India; 2 Department of Environmental Sciences, Jožef Stefan Institute, Jamova 39, Ljubljana 1000, Slovenia; 3 Amity Institute of Biotechnology, Amity University, Noida 201301, Uttar Pradesh, India

**Keywords:** chitosan, Iron oxide nanoparticles, metastasis

## Abstract

Compared with other nanomaterials, surface-modified iron oxide nanoparticles (IONPs) have gained attraction for cancer therapy applications due to its low toxicity, and long retention time. An innocuous targeting strategy was developed by generation of fluorescein isothiocyanate (FITC)-labeled peptide (growth factor domain (GFD) and somatomedin B domain (SMB)) functionalized, chitosan-coated IONPs (IONPs/C). It can be used to target urokinase plasminogen activator receptor (uPAR), which is a surface biomarker, in ovarian cancer. Binding affinity between uPAR and peptides (GFD and SMB) were revealed by *in-silico* docking studies. The biophysical characterizations of IONPs, IONPs/C, and IONPs/C/GFD-FITC or SMB-FITC nanoprobes were assessed via Vibrating Sample Magnetometer (VSM), Transmission Electron Microscopy (TEM), Dynamic Light Scattering (DLS), and Fourier Transform Infrared Spectroscopy (FT-IR). Prussian Blue staining, fluorescence spectroscopy, and fluorescence imaging were performed to confirm the targeting of nanoprobes with the surface receptor uPAR. The combination of IONPs/C/GFD+SMB showed efficient targeting of uPAR in the tumor microenvironment, and thus can be implemented as a molecular magnetic nanoprobe for cancer cell imaging and targeting.

## Introduction

Cancer remains one of the leading causes of mortality even though substantial advancement in research over the last decades has taken place [1]. Overexpressing surface receptors are ideal marks for targeting and imaging of cancer cells. To develop efficient imaging probes high affinity peptides need to be selected as the targeting agent [2]. Urokinase Plasminogen Activator Receptor (uPAR) that plays a significant role in tissue remodeling, embryogenesis and wound healing is overexpressed in human cancers such as tumors, leukemias, lymphomas indicating invasion and metastasis [3–7]. uPAR belongs to a family of proteins called the lymphocyte antigen 6 which have a globular structure consisting of five to six antiparallel β-strands linked via four to five disulfide bonds [8,9]. It comprises three domains; D1, D2, D3 packed together and the central region involves the residues from three domains that binds with uPA at the amino terminal growth factor domain (GFD). The vitronectin (Vn)-binding site is present at a linker sequence which connects domain D1 and D2 of uPAR that attached to the N-terminal of somatomedin B (SMB) domain present in Vn [10]. Since, the uPA- and Vn-binding sites are discrete, uPAR can bind to GFD and SMB ligands by allosteric modulation (a group of ligands that bind with a receptor for conformational changes in response to a stimulus) [11]. Currently uPA and plasminogen activator inhibitor-1 (PAI-1) are identified as the biomarkers for tumor by the American Society of Clinical Oncology as prognosticators of tumor recurrence [12]. Studies have shown that a high level of uPA in tumor tissues could be a powerful prognostic marker for breast cancer [13,14].

Several biomolecules such as antibodies, and peptides conjugated with nanoparticles (AuNPs, IONPs, Hollow particles, mesoporus silica) have been labeled using fluorescent dyes for efficient targeting and imaging [15–18]. Peptides have proved to be ideal for targeting due to its low cell toxicity, rapid renal clearance pharmacokinetics, small size, and ease of synthesis or modification, high specificity, accumulation in specific tissues for image-guided diagnosis, and efficient delivery to tumor cells [19]. It has been reported that specific peptides recognize the tumor and cancer cells more effectively for cancer therapy. In a recent study, biocompatible AuNPs labeled with fluorescent dye and functionalized with U11 peptide were used in targeting of uPAR [20]. New treatment strategies based on iron oxide nanoparticles (IONPs) have tumor diagnostic and therapeutic potential [21–24]. The presence of Fe group has affirmed its magnetic properties and alternative to gadolinium as a contrast agent [25,26]. IONPs can be suitably surface modified with low cellular cytotoxicity and long blood retention time making it an ideal entrant for imaging as well as therapeutics [27,28]. However, IONPs tendency to aggregate in a complex biological environment due to its magnetic properties are prone to alterations. Thus, IONPs were modified with polymers or surfactants such as chitosan, polyethylene glycol and poly-(maleic anhydride-alt-1-octadecene) and lactoferrin to provide stability and improve bioavailability [29–33]. Usage of chitosan polymer (C) is an added advantage because of its biodegradable nature, positive charge, pH responsiveness, and less toxicity that makes it a suitable vehicle for targeting, and drug delivery of peptides, drugs, or proteins [34–38].

In the present study, we hypothesized binding of GFD and SMB peptides with uPAR in an allosteric modulation (where binding of GFD ligand promotes association of SMB ligand in a close proximity). To test this hypothesis, we have used chitosan functionalized IONPs that were further labeled with FITC-GFD and FITC-SMB peptides and conducted various experiments to observe efficient imaging of uPAR-overexpressing cells by nanoprobe uptake, Prussian Blue staining and fluorescence microscopy. The IONPs were synthesized, coated with chitosan polymer, and conjugated covalently with peptides (GFD and SMB) in a successive way for efficient uPAR imaging. GFD and SMB peptide sequences were linked with fluorescein isothiocyanate (FITC) at N-terminal. The carboxyl group of both the peptides were attached with the free amine group of IONPs/chitosan (NH_2_–) via carbodiimide activation [16,39–40]. The IONPs coating, and labeling step was well characterized by UV-Vis spectroscopy, Dynamic Light Scattering (DLS), Fourier Transform Infrared Spectroscopy (FT-IR), Vibrating Sample Magnetometer (VSM), and Transmission Electron Microscopy (TEM), and further analyzed *in-vitro* by Prussian Blue staining, fluorescence spectroscopy and microscopy for uPAR-specific targeting and imaging. Cytotoxicity was measured by MTT (3-(4,5-dimethylthiazol-2-yl)-2,5-diphenyltetrazolium bromide) method. The research work carried out showed that chitosan-coated IONPs (IONPs/C)/GFD+SMB nanoprobe uniquely binds more proficiently in uPAR-overexpressing cells. Therefore, the established nanoprobe can be cast-off as an efficient technique for selective uPAR receptor targeting in tumor imaging.

## Materials and methods

### Apparatus

Hydrodynamic diameter and zeta potential of samples were verified via DLS (Anton Paar). Morphology and particle size were observed in TEM (JEOL-JEM 2010) using accelerating voltage of 200 kV. Magnetic properties of particles were observed using VSM (Lakeshore 665). Chemical modification of the IONPs and its conjugates were analyzed by Fourier Transform Infrared Spectroscopy (Nicolet iS20 FTIR Spectrometer). Fluorescence graph of samples were measured using PerkinElmer (*EnSpire*® *Multimode Plate Reader).* Images of Prussian Blue staining and fluorescence imaging were taken on Zeiss inverted fluorescence microscope (Axio observer 7).

### Reagents

Iron oxide (II, III) nanoparticles, Chitosan, N-(3-Dimethylaminopropyl)-N′ethylcarbodiimide hydrochloride (EDC), N-hydroxysuccinimide (NHS), Glacial acetic acid, and *In-Vitro* Toxicology Assay Kit (MTT based) were procured from Sigma–Aldrich, India. Monosodium phosphate (NaH_2_PO_4_), disodium phosphate (NaH_2_PO_4_), tris base, sodium chloride (NaCl), sodium hydroxide (NaOH), sodium bicarbonate (NaHCO_3_), sodium carbonate (Na_2_CO_3_), Potassium hexacyano ferrate (II) trihydrate, and glycine were purchased from Sisco Research Laboratories (SRL, India). GFD and somatomedin B domain (SMB) peptides were synthesized and purified as mentioned previously [41] and aliquots were stored at −20°C. Hydrochloric acid (HCl), nitric acid (HNO_3_), and glacial acetic acid of analytical grade were obtained from Fisher Chemical. Dulbecco’s modified Eagle’s medium (DMEM), penicillin, streptomycin, and 0.25% trypsin-EDTA (ethylenediamine tetraacetic acid) were bought from Gibco Laboratories (India). Fetal Bovine Serum (FBS) was acquired from Himedia Laboratories (India). Nunc microtiter plates 96-well were procured from Nunc, India. Carbon-coated copper TEM grids were bought from Ted Pella Inc. (Redding, Canada). Ovarian cancer cell line (SKOV3) was gifted from CSIR-IICB, Kolkata. All reagents were of high analytical grade and buffers were made in double distilled water unless mentioned otherwise. All experiments were independently carried out three times and at room temperature unless specified otherwise.

### *In-silico* analysis

#### Docking studies of peptides

The experimentally determined peptide sequences of GFD and SMB were downloaded from Uniprot database [42]. The RCSB Protein Data Bank (https://www.rcsb.org/) was used to obtain the uPAR (1YWH) receptor protein structure in PDB format. The peptide 3D structures were designed by a *de novo* method using PEP-FOLD3 online tool [43]. The structural compatibility and binding interaction of SMB and GFD peptides with uPAR was further studied. Docking of Protein–Peptide were carried out by using the ClusPro 2.0 server [44]. The hydrogen bonds and the root-mean-square deviation (RMSD) values were measured using Chimera software [45], Pymol visualization tools, and the authenticity of the hydrogen bonds were confirmed using LIGPLOT software [46].

#### Molecular dynamics simulation of uPAR receptor/peptide complex

To study the dynamic behavior of uPAR, uPAR-GFD, uPAR-SMB, and uPAR-GFD-SMB complex, the GROMACS 2020.3 [47] package was used to run a molecular dynamics (MD) simulation for 50 nanoseconds (ns). For apo (unbound protein) and complexes, the OPLS-AA/L all-atom force field was applied to obtain the topology. The docked complex was positioned in the central point of a cube, with the periodic boundary condition (PBC) set to 1 Å from each box side. A simple point charge (SPCE) water model was used to surround the protein/protein complexes within the cube, and the system was made neutral by replacing equivalent numbers of water molecules with the same number of counter ions (Na^+^ or Cl^−^). To minimize the energy of the system, a steepest descent technique was used for 50000 steps with 1000 kJ/mol/nm maximum force applied, and the same parameters were continued for the complex. All results were analyzed using Xmgrace and VMD tools.

### Preparation and characterization of IONPs/C

IONPs were commercially purchased and the surface of IONPs was coated with chitosan (IONPs/C) to reduce its tendency to aggregate in a biological environment. Chitosan solution was dissolved in 1% (v/v) glacial acetic acid and was kept stirring continuously for 3–4 h at 28–30°C while pH 5.6 was maintained by addition of sodium hydroxide (NaOH) solution. Chitosan polymer (C) and IONPs were sonicated and mixed in such a way that one part of IONPs was fixed and with variable ratio of the polymer (10, 20, 30, 40 and 50) to study the role of polymer coating on IONPs. The mixture was stirred overnight (O/N) at 37°C, and sonicated for 30 min at 37°C to ensure the monodispersity and characterized by DLS to verify the hydrodynamic diameter, polydispersity index (PDI) and zeta potential, TEM to confirm the average particle size, FT-IR to determine the functional groups, and VSM to analyze the magnetic properties of the particles.

### Labeling of IONPs/C with uPAR targeting peptides

uPAR targeting peptides were obtained and synthesized via FMOC method previously described in the work carried out by Shahdeo et al. [41] Synthesized GFD and SMB were conjugated on to the coated IONPs/C via EDC/NHS carbodiimide coupling method, where EDC acts as a linker to couple the amine group of chitosan of IONPs/C to the C-terminal of peptides. IONPs/C remained stable and provided a reactive amine group on the particle surface for bioconjugation. This amine group was used to form an amide bond with the carboxyl group of peptides. The stock solution (1 mg/ml) of both EDC and NHS was prepared in 1× Phosphate Buffer Saline (PBS) pH 7.4. Seventy-five microliters of both the EDC and NHS was added dropwise to different dilutions of the peptides (2.0, 1.5, 1.0, 0.5 µg/ml) and incubated for at RT for 1 h and further IONPs/C were mixed in the solution and kept at 4°C O/N. Conjugated peptide complexes (IONPs/C-GFD or SMB) were further characterized to confirm the functionalization.

### Stability studies of peptide functionalized IONPs/C at different pH

The stability of the IONPs/C functionalized with GFD and SMB peptides were evaluated at three different pH 3.0, 7.0 and 9.0 and characterization was done using UV-Vis spectroscopy, and DLS for hydrodynamic diameter and zeta potential analysis. Glycine-HCl (0.1 M glycine, 1 N HCl, pH 3.0), phosphate buffer (PB) (75 mM monosodium phosphate (NaH_2_PO_4_), 24 mM disodium phosphate (NaH_2_PO_4_) pH adjusted with HCl/NaOH to 7.4) and carbonate buffer (0.2 M solution of sodium carbonate, 0.2 M solution of sodium bicarbonate, pH 11.0) were used to analyze the stability of the conjugates. Peptides were incubated in the same buffer as that used for functionalization and further resuspended in the respective buffers of different pH.

### Cellular uptake of uPAR targeting peptides

#### Fluorescence spectroscopy

To determine the fluorescent uptake of targeting peptides, 10000 cells were grown in 96-well plate and treated with IONPs/CG, IONPs/CS, IONPs/CGS in DMEM comprising 10% FBS and 1% P/S. After 24 h, the cells were washed three times for 5 min to remove the unbound nanoparticles. One hundred microlitres of DMEM was added, and fluorescence was analyzed with excitation at 488 nm, while emission was observed between 500 and 600 nm.

#### Prussian Blue staining

Prussian Blue staining was carried out to observe the presence of iron content in the cells treated with IONPs. SKOV3 cells were cultured for 24 h at 37°C after which they were treated and incubated for 12 h with 1 µg/ml of IONPs, IONPs/C, IONPs/CG, IONPs/CS, and IONPs/CGS. Later, treated and untreated cells were washed with 1× PBS and fixed using 4% formaldehyde solution for 30 min. Formaldehyde-fixed cells were incubated with 5% potassium hexacyano ferrate(II) trihydrate and 10% HCl (1:1 mixture). The cells were incubated for half an hour and washed with 1× PBS to remove the extra staining solution and examined under the microscope. For each treatment, bright-field optical images were obtained and analyzed for the Prussian Blue staining.

#### Fluorescence microscopy

SKOV3 cells (1 × 10^4^ cells/well) were grown for 24 h at 37°C. The medium was changed, while, the culture medium was treated with IONPs (mentioned above) (10 μg/ml/well) and incubated for 24 h at 37°C. Subsequently, the cells were washed thrice with PBS (kept on ice) and fixed using 4% paraformaldehyde for 30 min, and again washed thrice with PBS. 4′,6-diamidino2-phenylindole (DAPI) was used for staining of the nuclei for 10 min and visualized under a fluorescence microscope.

### *In-vitro* cytotoxicity study

The cell toxicity effect of both bare IONPs and its peptide formulations were evaluated on SKOV3 cells with the standard MTT assay. SKOV3 cells (1 × 10^4^ cells/well) were plated in 96-well culture plates. After culturing for 24 h, the cells were treated with IONPs and various conjugates for 24 h, following which 20 μl of 5 mg/ml MTT solution was added. After 4 h of incubation, the medium was discarded, and 150 μl of dimethyl sulfoxide (solubilizing buffer) was added to the cells. The optical density was taken at 490 nm.

### Statistical analysis

For statistical analysis, all data have been expressed as mean ± standard deviation (SD). The graphs and curves were plotted in Graph-Pad Prism 5 and Microsoft Office Excel 2007.

## Results and discussion

### *In-silico* analysis

#### Docking studies of peptide with uPAR receptor

To assess how functionally stable the whole protein is, the uPAR–GFD complex was docked with SMB (whose binding affinity is −623 kcal/mol, is lesser than the docking studies of uPAR with GFD and SMB individually). The binding affinity was higher when uPAR was bound separately with GFD and SMB (−982.4 and −753.2 kcal/mol, respectively) and RMSD value of 0.2 and 0.1, respectively.

**Figure 1:**
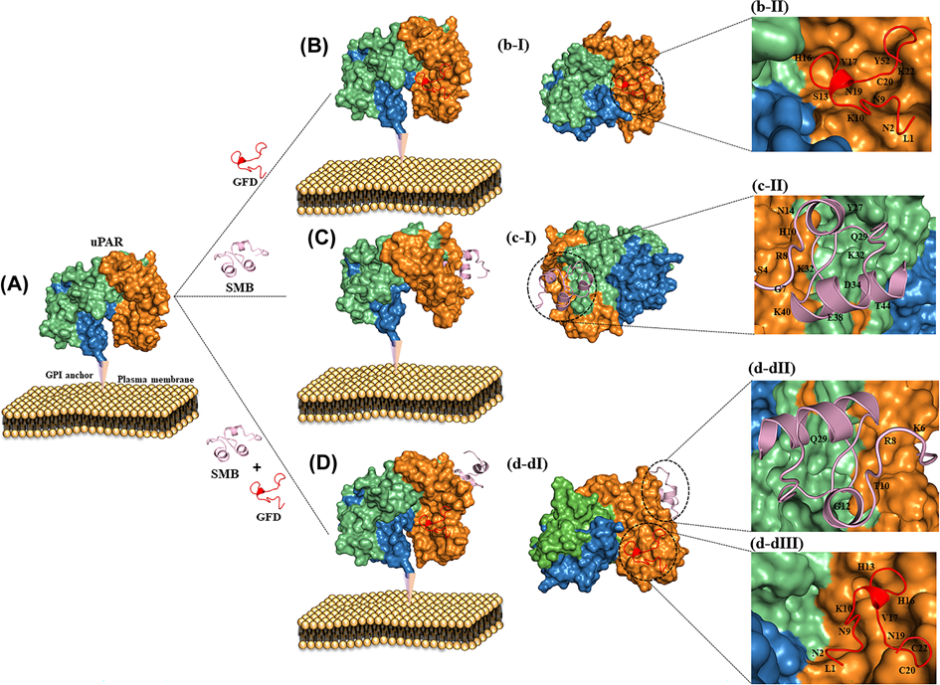
Docked poses of uPAR with GFD and SMB (**A**) uPAR attached with GPI anchored plasma membrane. (**B**) Surface pose of uPAR with GFD. (**B-I**) The binding pose of targeting peptide (GFD) (red ribbon pose) within the uPAR cavity domain DI (orange). (**B-II**) Interaction of GFD peptide directly with the uPAR DI central cavity. (**C**) Surface pose of uPAR interacting with SMB. (**C-I**) The binding pose of targeting peptide SMB within the uPAR cavity domain DII (lime green). (**C-II**) Interaction of SMB peptide (light plum ribbon pose) directly with the uPAR DII central cavity. (**D**) Surface pose of uPAR interacting with both GFD and SMB. (**D-I**) Both the targeting peptides interacting with different uPAR domains. (**D-II,III**) The dotted circle simultaneously visualizes the interaction of the central cavity with different GFD and SMB amino acid residues.

The binding affinity of the uPAR–GFD complex was significantly reduced when docked with SMB, with an RMSD value of 0.3 and a binding affinity of −623 kcal/mol, indicating a major conformational shift and energy difference in the structure of the uPAR/GFD/SMB complex (Figure 1). The docking results revealed that amino acid involved in binding of uPAR with peptides are (uPAR+GFD)-(D254-L1, H251-N2, D140-K10, S101-S13, S101-H16, E42-K20, D141-N9, T27-N19, E33-H16, T8-C20, N9-K22, Y57-H16, R142-V17)), (uPAR+SMB)- (S101-Q29, R142-Q29, R142-R8, S101-Y28, E33-Y35, E33-K45, K139-E23, S257-S4, D254-S4, D254-K56, Y57-T10, E230-K18, E230-Q20, E42-V15, E42-N14, N259-Q2)), (uPAR+GFD+SMB)-(N9-K22, T8-C20, L40-N19, E33-H16, Y57-H16, D141-N9, D140-K10, R142-V17, S101-H16, S101-H13, E42-C20, D254-L1, H251-N2; Y149-K6, N52-R8, N52-T10, E119-G12, S81-Q29)) and complexes formed were found to be very stable.

#### MD simulation

MD simulations aim to mimic the real behavior of protein molecules in their environment, taking into account their flexibility and particle movement over time, rather than the static image obtained through methods like crystallography [48]. Based on the results of docking analysis, MD simulations were performed with uPAR, uPAR-GFD, uPAR-SMB and uPAR-GFD+SMB models, and the dynamic behavior of the proteins was analyzed. According to the MD simulation review, the apo (unbound) form of uPAR displayed more RMSD fluctuations than complexes (Supplementary Figure S1A). The difference in RMSD was revealed by large conformational changes in uPAR. The RMSD analysis of the peptide complex structure remained more stable than the apo form throughout the simulation. The root mean square fluctuation (RMSF) values represent the thermodynamic stability and degree of movement of each residue. Smaller RMSF values indicate a more stable region, while larger RMSF values indicate a more flexible region. The regions with crucial interactions with uPAR showed fewer fluctuations, as shown in (Supplementary Figure S1B). The results from both the apo and docked complexes showed that upon peptide recognition, the residues in the peptide-binding regions stabilized. The radius of gyration (Rg) analysis reveals each molecule’s stability level [49] as well as the structure’s overall dimension [50]. After 30 ns of MD simulation, apo and complexes; uPAR, uPAR-GFD, uPAR-SMB and uPAR-GFD+SMB moved with relatively constant values of 2.11, 2.00, 1.99 and 2.13 nm, respectively, as shown in (Supplementary Figure S1c). These figures are very close to the uPAR average (2.11 nm). As a result, during the 50-ns MD simulation, the complexes remained extremely stable.

### Optimization of chitosan polymer IONPs ratio

**Figure 2:**
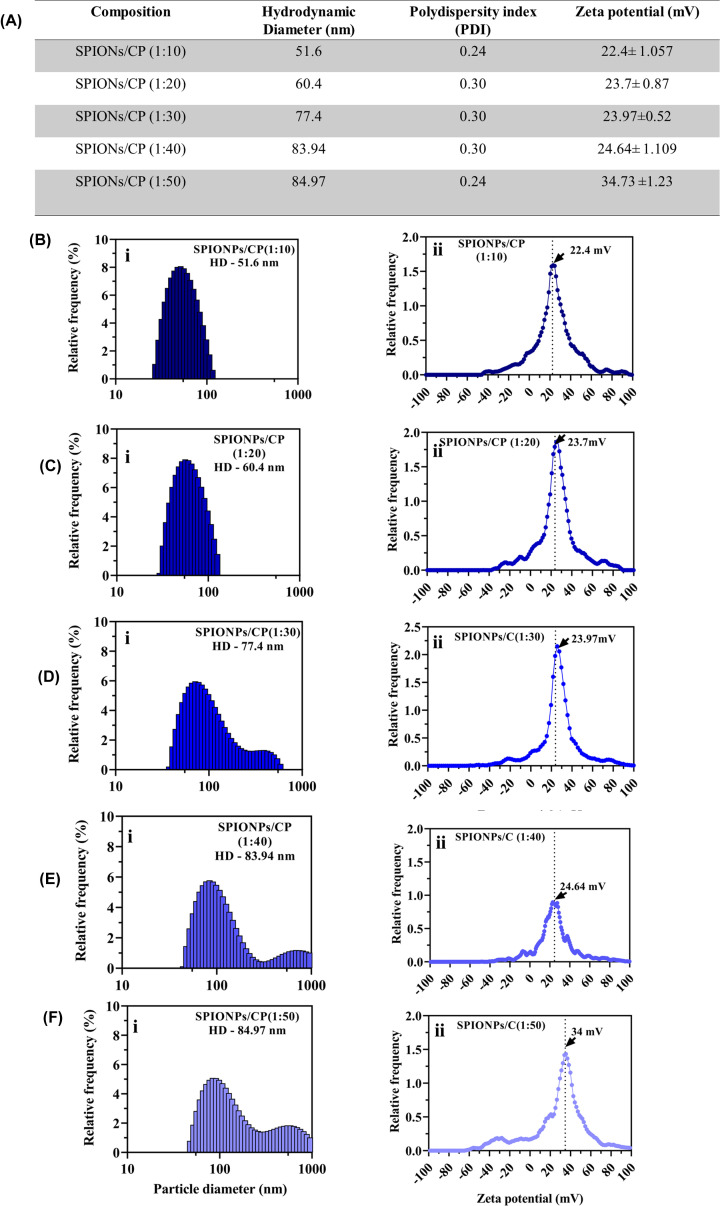
Optimization and characterization of different molar ratio of IONPs/C (**A**) Table representing the hydrodynamic diameter, PDI and zeta potential of different IONPs/C ratios. (**B–F**) (i) Hydrodynamic diameter where particle size increased from 51 to 60.4, 77.4, 83.94, and 84.97 nm with increase in the molar ratio to 1:50 and (ii) zeta potential of different ratios of IONPs/C 22 ± 1.057, 23 ± 0.87, 23 ± 0.52, 24 ± 1.109, 34 ± 1.23 respectively.

The different molar ratio of IONPs:C (1:10–1:50) was optimized and characterized by DLS. As shown in Figure 2, increase in the concentration of C, the particle size increases along with the PDI value; 1:10 ratio was standardized for all subsequent experiments as the optimum concentration for coating. Since it showed a low PDI value of 0.24 tending away from 1, which indicated the homogeneous state of the solution along with a stable zeta potential value of 22 ± 1.05. Hydrodynamic Diameter, PDI and zeta potential observed at different concentrations of IONPs/C was represented as a tabular value in (Figure 2A). Increase in the hydrodynamic diameter from 51 to 60.4, 77.4, 83.94, and 84.97 nm and zeta potential of different ratios of IONPs/C was found to be 22.4 ± 1.057, 23.7 ± 0.87, 23.97 ± 0.52, 24.64 ± 1.109, 34.73 ± 1.23 respectively, which showed the net positive charge on IONPs surface (Figure 2B–F).

### Characterization of IONPs, and IONPs/C GFD/SMB peptides

**Figure 3:**
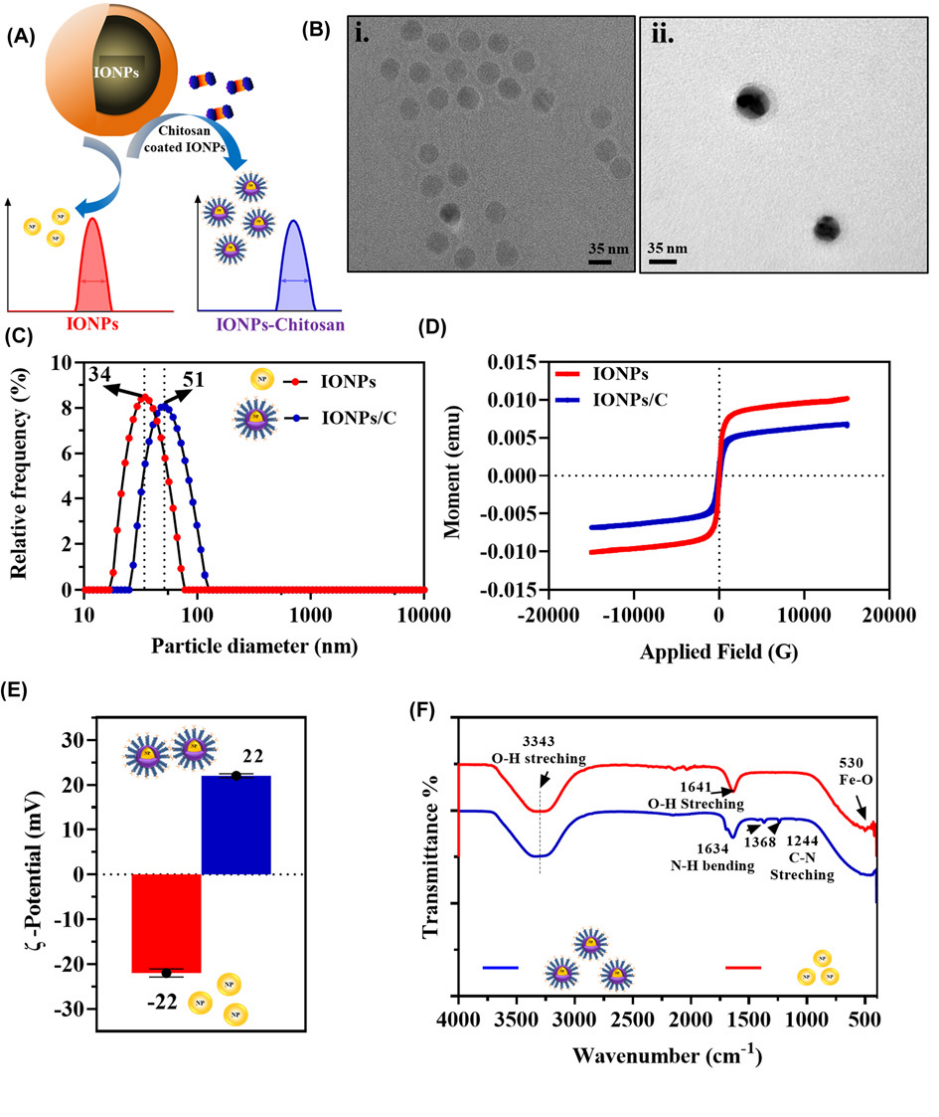
Characterization of IONPs, and IONPs/C (**A**) Surface modifications of IONPs with chitosan polymer (C). (**B**) TEM image of (i) bare IONPs and (ii) IONPs/C with average particle size of 35 ± 5 nm. (**C**) Hydrodynamic diameter of IONPs (34 nm), IONPs/C (51 nm) showed increase in size with addition of chitosan polymer. (**D**) Zeta potential of IONPs (−22 mV), IONPs/C (+22 mV) displayed increase in surface charge. (**E**) VSM hysteresis loops of IONPs, and IONPs/C showed shift in saturation magnetization value from 0.008 to 0.006 emu.G^−1^. (**F**) FT-IR spectrum of IONPs, and IONPs/C showing well-defined peaks of IONPs at 530, 1634 and 1244 cm^−1^ which depicts the Fe–O of iron oxide, N–H bending and C–N stretching of amine.

Figure 3A depicted modifications made to IONPs such as coating of polymer with chitosan and structure of amine functionalized IONPs. The average particle size of IONPs was at approximately 35 ± 5 nm with monodispersed state (Figure 3B,i) and IONPs coated with chitosan (Figure 3B,ii). Hydrodynamic diameter of IONPs, and IONPs/C were 34 and 51 nm respectively (Figure 3C) that confirmed successful coating of chitosan polymer. PDI values of all three tended away from 1 which showed homogeneous dispersion of the particles (Table 1). Zeta potential was measured to understand the changes in the surface charge. Zeta potential was −22 ± 0.4 and +22 ± 0.91 – for IONPs, and IONPs/C respectively (Figure 3D). Higher value of zeta potential indicated the existence of larger repulsive force between the particles which favors less aggregation and higher stability as perceived in IONPs/C. The VSM hysteresis loops showed a smooth M-H curve followed by saturation in case of IONPs and IONPs/C (Figure 3E). Magnetization of IONPs and IONPs/C was found to be 0.008 and 0.005 emu.G^−1^ respectively indicated saturation of magnetization in case of IONPs/C was reduced as compared with IONPs, confirmed successful polymer coating on the surface of bare IONPs [51]. Chemical interactions of IONPs and IONPs/C were determined by FT-IR (Figure 3F) and the characteristic Fe-O vibrational peak was observed in case of IONPs at 530 cm^−1^ [52]. Peaks positioned at 1641 and 3343 cm^−1^ (broad) were due to hydroxyl group of O–H stretching as the solvent was aqueous in all three cases. IONPs/C showed N–H bending and C–N stretching at 1634 and 1244 cm^−1^ [53], confirmed the addition of surface amine. In addition to that, the single peak observed at 1368 cm^−1^ corresponds to O–H bending.

**Table 1:** DLS characterization of IONPs and its nanoprobes

S.No.	Nanoprobes	Hydrodynamic diameter (nm)	PDI	ζ potential (mV)
**1.**	IONPs	34	0.26	−24
**2.**	IONPs/C	51	0.22	22
**3.**	IONPs/CG	65	0.27	21
**4.**	IONPs/CS	66	0.35	20
**5.**	IONPs/CGS	77	0.35	18

Hydrodynamic diameter values depicting the increase in particle size (34, 51, 65, 66, 77 nm) with additional coating and conjugation with uPAR targeting peptides along with PDI values. Zeta (ζ) potential value indicated the increase in positive charge on the surface after coating (−24, 22, 21, 20, 18 mV).

**Figure 4:**
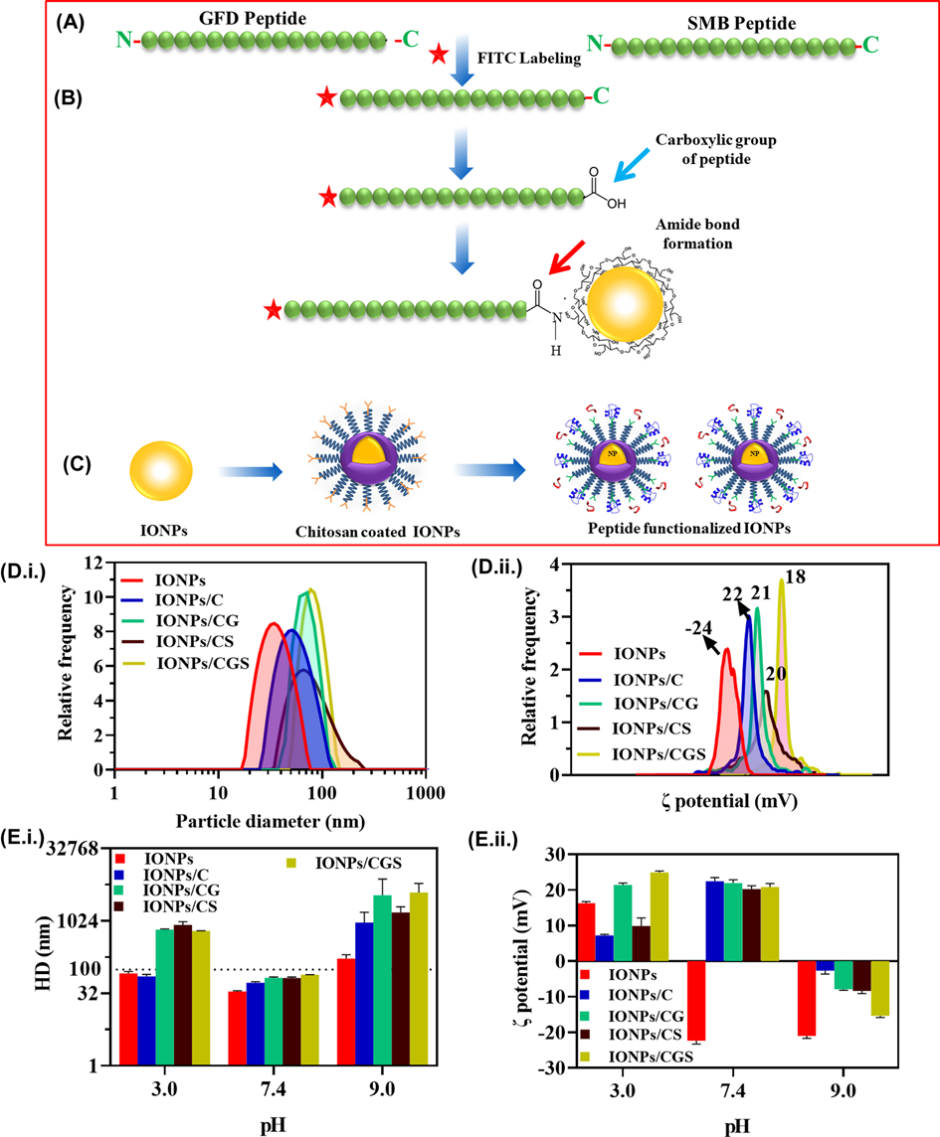
Schematic representation and characterization of peptide labeled nanoprobes (**A**) Sequence of the uPAR targeting peptides (GFD and SMB) derived from the ATF region of ligand uPA and Vn. (**B**) The predicted peptides were synthesized by FMOC method and FITC was linked to the N-terminal of GFD and SMB peptides. (**C**) Carbodiimide chemistry was used to conjugate the uPAR targeting peptide with IONPs/C. (**D**) (i) Hydrodynamic diameter (34, 51, 65, 66, 77 nm) and (ii) zeta potential of peptides functionalized with IONPs/C (−24, 22, 21, 20, 18 mV). (**E**) Stability of the nanoprobe at different pH: (E,i,ii) hydrodynamic diameter and zeta potential of IONPs, IONPs/C and GFD and SMB peptide conjugates at different pH.

The short peptide of amino-terminal fragment (ATF) of uPA and N-terminal SMB of Vn contained the uPAR binding region that was synthesized via FMOC method and FITC was linked at the N terminal of both the peptides. Synthesized peptides were further labeled with IONPs/C via carbodiimide coupling chemistry (EDC/NHS). The peptide sequence of GFD and SMB were depicted in (Figure 4A). Glutamic acid present at C terminal of peptide reacted with the amine group of chitosan, stemmed in an amide bond formation (Figure 4B,C). IONPs/C functionalized peptides have been denoted as IONPs/CG (G for GFD), IONPs/CS (S for SMB), and IONPs/CGS (G+S for GFD+SMB). After successful labeling, peptide nanoprobes were analyzed by DLS to measure the hydrodynamic diameter that showed polymer-coated IONPs of size 51 nm increased to 65–66 nm, and 77 nm after labeling with GFD, SMB and GFD+SMB respectively (Figure 4D,i). Zeta potential of the peptide conjugated nanoparticles increased from −24 to 18 mV, indicated increasingly, positive charge that confirmed the successful conjugation of peptides with IONPs/C (Figure 4D,ii). The hydrodynamic diameter, PDI, and zeta potential of all the nanoprobes have been revealed in Table 1. Henceforth, IONPs/C showed superior characteristics for labeling of peptides that can be used further for *in-vitro* studies in uPAR-overexpressing cancer cells.

### Effect of pH on the stability of IONPs/C nanoprobes

Stability of the nanoprobe is a crucial step for efficient targeting and imaging, therefore different pH range (3.0, 7.4, and 9.0) were chosen to comprehend the stability at variable pH. Figure 4E,i,ii showed hydrodynamic diameter and zeta potential of peptides conjugated with IONPs/C respectively. Labeling of peptides leads to increase in the particle size. However, at pH 3 (78–637 nm) and pH 9.0 (168–3980 nm), the nanoprobes showed aggregation due to decrease in the electrostatic repulsion making them unstable. On the contrary, the surface charge of nanoprobes increased (net positive charge) at pH 3 and 7.4. However, at pH 9 the net negative charge was noticed, possibly due to alkaline solution. Hereafter, pH 7.4 was selected as the optimum pH for further studies of the nanoprobes. Supplementary Table S1 depicted the numerical values of hydrodynamic diameter and zeta potential.

### Nanoprobes uptake assay and internalization studies

**Figure 5:**
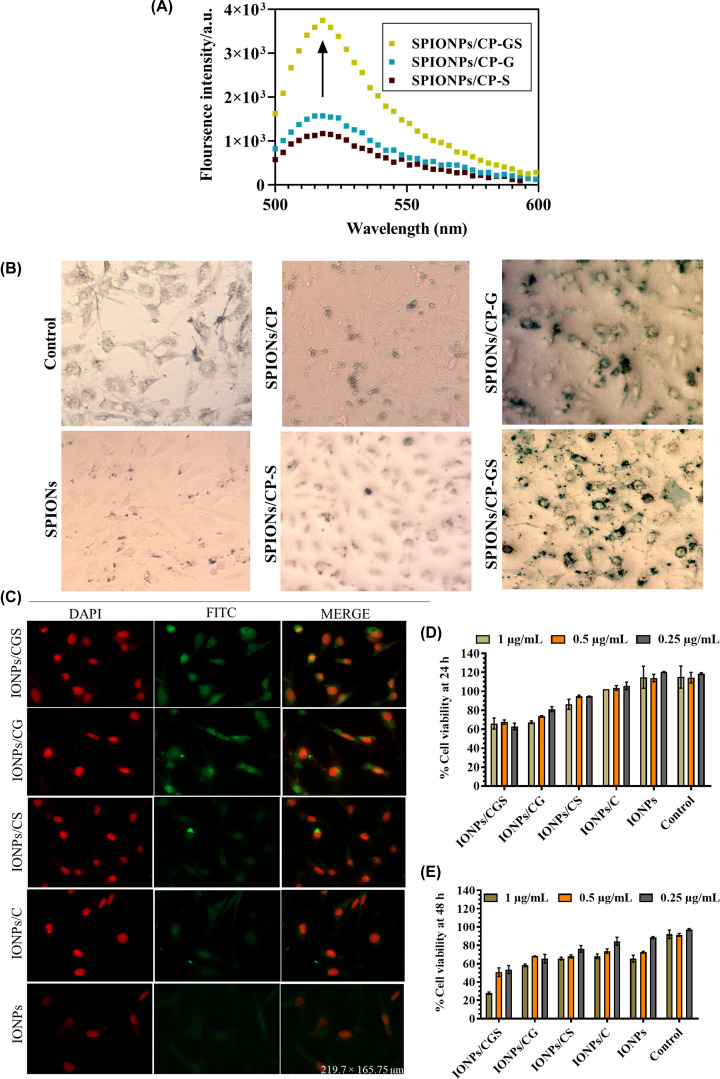
Nanoprobes uptake assay and Prussian Blue staining (**A**) Fluorescence uptake assay measurement after treatment of uPAR overexpressing SKOV3 cells with the different peptide nanoprobes. (**B**) Prussian Blue staining of SKOV3 cells treated with the IONPs, IONPs/C, IONPs/CS, IONPs/CG, IONPs/CGS, showed non-specific iron staining with unconjugated bare particles than IONPs/CGS, and IONPs/CG (scale bar 20 µm, magnification 40×). (**C**) Cellular uptake of peptide functionalized with IONPs with fluorescence microscopy; peptides linked with FITC (green) were co-localized on the surface of uPAR overexpressing SKOV3 cells. DAPI (red) was used to stain the nuclei (scale bar 20 µm, magnification 40×). (**D** and** E**) MTT assay at different concentration of nanoprobes at (i) 24 and (ii) 48 h. Controls were not provided with any treatment. The results were articulated as mean values ± SE of three independent experiments.

To determine the uptake efficiency of targeting peptide, SKOV3 cells were incubated with the different nanoprobes (IONPs, IONPs/C, IONPs/CG, IONPs/CS and IONPs/CGS), the fluorescence spectra were measured in the range of 500–600 nm, along single emission maxima at 516 nm. Higher gradation of nanoprobe uptake was observed in IONPs/CGS > IONPs/CG > IONPs/CS suggested that IONPs/CGS showed more efficient targeting and imaging in uPAR overexpressing cells (Figure 5A). Prussian Blue is a synthetic color used as a histochemical stain used to detect the presence of iron content in the cells after incubation with IONPs and peptide-conjugated IONPs. Furthermore, uptake studies were confirmed by Prussian Blue staining. The cells were incubated with non-targeted IONPs and IONPs functionalized with targeting peptides for 12 h and treated with Prussian Blue staining solution (Figure 5B). Cells incubated with IONPs retained a lighter stain, while cells treated with the uPAR targeting peptides showed more intense staining (IONPs/CGS > IONPs/CG > IONPs/CS). This observation suggested that uPAR targeting peptide functionalized IONPs/C can bind and internalize more efficiently than bare IONPs via receptor–ligand interactions in a cooperative manner.

To confirm the cellular localization events of nanoprobes by uPAR overexpressing cells, we accompanied fluorescence imaging experimentations. Figure 5C demonstrated the targeting and internalization of IONPs/CG, IONPs/CS, IONPs/CGS, IONPs/C, IONPs nanoprobe complex. The active engrossment of receptor-mediated targeting in incident of IONPs/CGS and IONPs/CG nanoprobes was witnessed in SKOV3 cells. Insignificant internalization was manifested in case of IONPs/CS, IONPs/C, IONPs. Furthermore, this observation supported the hypothesis that chitosan polymer improves the sustainability and stability of the nanoprobes, and combination of GFD and SMB complex is highly efficient for targeting and imaging of uPAR overexpressing cancer cells. In order to assess the cytotoxicity, *in-vitro* characterization of peptide nanoprobes were done. Measurement of cellular toxicity is highly significant for usage of peptides as an imaging probe. MTT assay is a conventional technique, which depends on color change of MTT by mitochondrial succinate dehydrogenase. SKOV3 cells were incubated with different concentrations of peptide nanoprobes and assessed using MTT assay. To estimate the cell viability, uPAR overexpressing SKOV3 cells were incubated with different concentrations nanoprobes (1 to 0.25 μg/ml) for the time interval of 24, 48 h and treated with MTT. Figure 5D and Figure 5E represented the cell viability of IONPs/C at 24 and 48 h with different nanoprobes. The cell viability pattern was observed in SKOV3 cells treated with IONPs/CGS > IONPs/CG > IONPs/CS at 24 h (Figure 5D) that decreased after 48 h at 1.0 μg.ml^−1^ upon extended incubation time (Figure 5E) which is due to selective targeting of IONPs/CGS > IONPs/CG in uPAR overexpressing cells. Though, IONPs and IONP/C treated cells showed significant cell viability in both cases (24 and 48 h). Thus, these results indicated that peptide nanoprobes showed efficient targeting on uPAR overexpressing cancer cells, while bare IONPs and IONPs/C have no substantial effect and can be utilized as a safe and efficient imaging tool in the future.

## Conclusions

In the present study, we have described the potential of uPAR targeting peptide as a powerful imaging probe coupled with polymer coated magnetic IONPs for selective targeting and efficient imaging in uPAR overexpressing cancer cells. Peptides obtained from the ATF region of uPA and Vn were recognized as a targeting tool and functionalized with IONPs to improve their stability and biocompatibility. Herein, the designed peptides GFD and SMB nanoprobes stimulated receptor-mediated targeting and cellular localization. Modified peptides were conjugated via carbodiimide chemistry with chitosan polymer coated with IONPs showed excellent stability that emphasize the crucial role of polymer. The projected hypothesis showed that the developed peptide nanoprobes (GFD+SMB) displayed high binding affinity for uPAR receptor by fluorescence-based uptake assays, Prussian Blue staining, and fluorescence microscopy above GFD or SMB peptides alone, creating a suitable target. Therefore, the above findings suggested that the polymer-coated stable IONPs are highly efficient and can be used as a powerful probe for imaging of cancer cells via receptor-mediated targeting.

## Supplementary Material

Supplementary Figure S1 and Table S1

## Data Availability

Data may be provided by authors upon reasonable request.

## Abbreviations

ATF: amino-terminal fragment
DLS: dynamic light scattering
DMEM: Dulbecco’s modified Eagle’s medium
EDC: N-(3-Dimethylaminopropyl)-N′ethylcarbodiimide hydrochloride
FBS: fetal bovine serum
FITC: fluorescein isothiocyanate
FT-IR: Fourier transform infrared spectroscopy
GFD: growth factor domain
IONP: iron oxide nanoparticle
IONPs/C: chitosan-coated IONPs
MD: molecular dynamics
MTT: 3-(4,5-dimethylthiazol-2-yl)-2,5-diphenyltetrazolium bromide
NHS: N-hydroxysuccinimide
O/N: overnight
PBS: phosphate buffer saline
PDI: polydispersity index
RMSD: root-mean-square deviation
SMB: somatomedin B domain
TEM: Transmission Electron Microscopy
uPAR: urokinase plasminogen activator receptor
Vn: vitronectin
VSM: Vibrating Sample Magnetometer
