# Supplementary material for: Polymeric biocompatible iron oxide nanoparticles labeled with peptides for imaging in ovarian cancer
Source: Biosci Rep. 2022 Feb 11;42(2):BSR20212622. doi: 10.1042/BSR20212622 (PMC8837818; doi:10.1042/BSR20212622)
Supplement: Supplementary Figure S1 and Table S1 [file BSR-2021-2622_supp.pdf]

Supplementary information

Polymeric biocompatible iron oxide nanoparticles labeled with peptides for imaging in ovarian cancer

Deepshikha Shahdeo[a], Akanksha Roberts[a], Veerbhan Kesarwani[a], Milena Horvat[b], Raghuraj Singh Chouhan[b, #], Sonu Gandhi[a,c,#]

[a]*DBT- National Institute of Animal Biotechnology (DBT-NIAB), Hyderabad-500032, Telangana, India.*

[b]*Department of Environmental Sciences, Jožef Stefan Institute, Jamova 39, 1000 Ljubljana, Slovenia.*

[c]*Amity Institute of Biotechnology, Amity University, Noida-201301, Uttar Pradesh, India.*

#Corresponding author:
E-mail: raghuraj.singh@ijs.si, Tel: +383 1 588 5268
E-mail: gandhi@niab.org.in, sonugandhi@gmail.com, Tel: +91 40 23120127

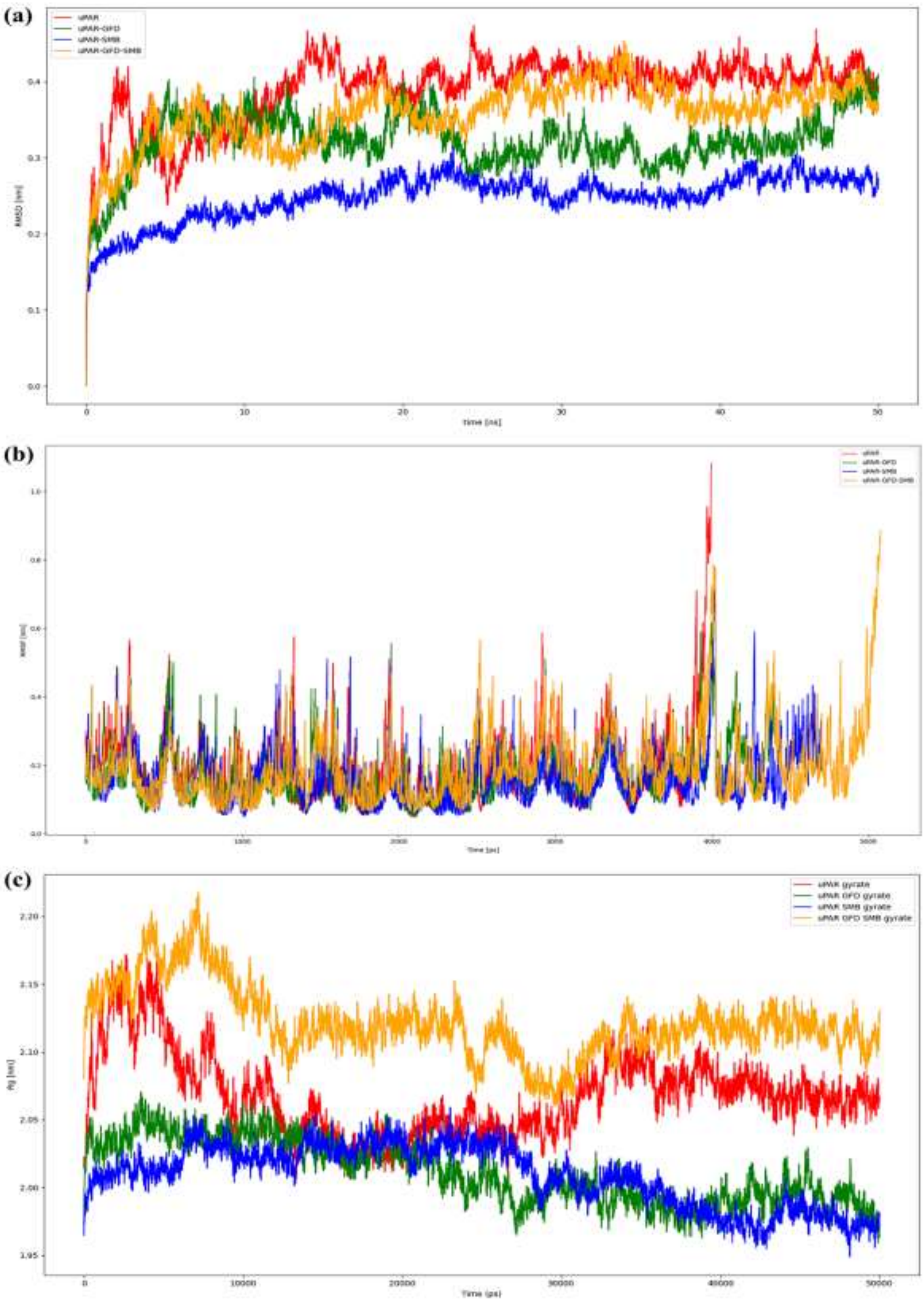

Fig. S1. Result from 50 nano second (ns) molecular dynamics simulation. **(a)** RMSD fluctuation, **(b)** Residual RMSF, **(c)** Radius of gyration (Rg).

Table S1. (a.i.) Hydrodynamic diameter and **(a.ii.)** zeta potential of different nanoconjugates of IONPs.

a.i

Sample	pH 3	pH 7.4	pH 9
IONPs	78	34	168
IONPs/C	77	51	945
IONPs/C-G	659	65	3478
IONPs/C-S	940	66	1527
IONPs/C-GS	637	77	3980

a.ii

Sample	pH 3	pH 7.4	pH 9
IONPs	16.3	-24	-21
IONPs/C	7.2	22	2.7
IONPs/C-G	21.4	21	-7.86
IONPs/C-S	9.9	20	-8.4
IONPs/C-GS	24.9	18	-15.35
